# Broad Spectrum Anti-Quorum Sensing Activity of Tannin-Rich Crude Extracts of Indian Medicinal Plants

**DOI:** 10.1155/2016/5823013

**Published:** 2016-04-14

**Authors:** Varsha Shukla, Zarine Bhathena

**Affiliations:** ^1^Department of Microbiology, Ramnarain Ruia College, Matunga, Mumbai 400019, India; ^2^Department of Microbiology, Bhavan's College, Andheri, Mumbai 400058, India

## Abstract

Quorum sensing (QS) mechanisms have been demonstrated to have significance in expression of pathogenicity in infectious bacteria. In Gram negative bacteria the autoinducer molecules that mediate QS are acyl homoserine lactones (AHL) and in Gram positive bacteria they are peptides called autoinducing peptides (AIP). A screening of tannin-rich medicinal plants was attempted to identify extracts that could interrupt the QS mechanisms in both Gram positive and Gram negative bacteria over a wide range of concentrations and therefore potentially be potent agents that could act as broad spectrum QS inhibitors. Six out of the twelve Indian medicinal plant extracts that were analyzed exhibited anti-QS activity in* Chromobacterium violaceum* 12472 and in* S*.* aureus* strain with* agr*:*blaZ* fusion over a broad range of subinhibitory concentrations, indicating that the extracts contain high concentration of molecules that can interfere with the QS mechanisms mediated by AHL as well as AIP.

## 1. Introduction

Unlike the early views on unicellular bacteria that suggested solitary behavior and little interactive capacity within a population, recent developments in ecological sciences suggest that communities, rather than individuals, play a major role in the maintenance of ecological stability [1, 2]. Explorations for cooperative behavior in microbial communities have recently established that bacteria do communicate to coordinate the behavior of the population. One such phenomenon of organized social behavior in unicellular organisms is called quorum sensing (QS) [2]. Quorum sensing is a process of communication in bacteria via which bacteria are able to sense whether they have reached a density corresponding to their quorate population. At this density they are able to alternate gene expression such that the phenotype will be able to sustain the activities best suited for survival in the new environment [3]. QS takes place when the concentration of autoinducers released by bacteria reaches a critical threshold concentration, at which they bind to receptors and activate them to trigger genes that encode information associated with several characteristics, like bioluminescence, plasmid conjugation, biofilm formation, toxin production, exopolysaccharide production, siderophore synthesis, sporulation, and motility [4, 5], several of which determine pathogenicity of the organism. In Gram negative bacteria the autoinducing molecules are commonly* N*-acylated homoserine lactones (AHLs) whereas in Gram positives they are small peptides called autoinducing peptides (AIPs) [6]. QS in most Gram negative bacteria takes place via the LuxI/LuxR system.* luxI* encodes an autoinducer synthase that catalyzes the formation of the signal molecule, AHL, that on reaching the quorum activates* luxR* that codes for the receptor of AHL. This complex then binds to the QS regulated promoters and leads to transcription of all genes controlled by QS system [3, 4, 7, 8]. The best studied QS gene in Gram positive bacteria is the* agr* locus in* Staphylococcus aureus* that codes for its pathogenicity determinants. The* agr* locus consists of two divergent operons that are activated by promoters P2 and P3, respectively. P2 acts as a promoter for genes that synthesize the autoinducer. P3 codes for RNAIII, which is the regulatory effector of the* agr* regulon and initiates the transcription of genes that encode a variety of exoproteins responsible for pathogenicity [6, 9–11].

Because QS controls the virulons in many infectious bacteria like* Vibrio cholerae*,* Escherichia coli*,* Bacillus cereus* [6, 12],* Pseudomonas aeruginosa*,* Staphylococcus aureus* [3], and* Acinetobacter baumannii* [13], blocking of this mechanism would prevent pathogenicity and hence attenuate the infectious agent [12]. Quorum sensing inhibitors (QSI) can be excellent antipathogenic agents that will not interfere with growth but can prevent pathological consequences. Virulence determinants in pathogens are not strictly essential for viability. Thus anti-QS compounds will not induce mutagenesis that can lead to the emergence of resistance. Such agents will instead promote clearance of the pathogen by stimulating the immune mechanisms with live, multiplying organisms for a longer duration [14]. Rasmussen and Givskov (2006) [15] have suggested that quorum sensing can be attacked at three points: either by blocking production of the autoinducer, by inactivating it, or by interfering in its binding to the receptor.

In the recent years, screening for anti-QS substances in eukaryotes like plants, algae, and fungi is gaining significance. As these eukaryotes do not possess active immune systems, they rely on physical and chemical defense mechanisms. It is therefore hypothesized that secondary metabolites produced by them can aid in preventing colonization by pathogens. Such natural quorum sensing interfering compounds are reported in exudates of several vegetables and spices and are also found to be secreted by* Penicillium* spp. and* Chlamydomonas reinhardtii* [4, 14–17].

Considering the enormous number of plant varieties and the chemical diversity that plants inherently possess, screening plants for medicinally significant compounds seems rational. Various medicinal plants have been screened for their anti-QS potential [18–22], and several phytochemicals have been shown to affect expression of pathogenicity via disruption of QS [23–27]. The current study uses an ethnobotanical approach and focuses on anti-QS properties of tannin-rich extracts of plants used in Indian traditional and folk medicine, as plants rich in tannins are naturally protected from predation and pathogens. Antimicrobial, antiviral, and anticancer activities of tannins have been reported [28, 29]. Anti-AHL activity of synthetic tannic acid was reported by Huber et al. (2003) [30]. Recently some reports on purified tannin-rich components exhibiting either anti-AHL or anti-*agr* activity have also been made [31–34]. In the current study, we have quantified both anti-AHL and anti-*agr* activity of tannin-rich plant extracts with the aim of determining spectrum of activity and of suggesting their potencies as anti-QS substances.

## 2. Materials and Methods

### 2.1. Preparation of Plant Extracts

Powders of seeds of* Syzygium cumini* and* Embelia ribes* were acquired from the local market. Fruits of* Phyllanthus emblica*,* Terminalia bellirica*, and* Terminalia chebula*; pericarp of* Punica granatum*; flowers and seed kernel of* Mangifera indica*; and the barks of* Acacia arabica*,* Terminalia arjuna*,* Thespesia populnea*, and* Casuarina equisetifolia* were collected from Mumbai and the respective plant samples were authenticated at Agharkar Institute, Pune, and The Blatter Herbarium, St. Xavier's College, Mumbai. The plant parts were washed, dried, and powdered. Methanolic extracts were prepared by soaking the powders (1 gm) in methanol (10 mL) overnight. These extracts were used for phytochemical screening. For the quantitative biological assays the dried methanolic extracts were dissolved in 7.8% DMSO to known concentrations and stored at −25°C [35].

### 2.2. Phytochemical Screening of Extracts

Extracts were qualitatively analyzed for presence of phytochemicals, namely, saponins, alkaloids, flavonoids, di- and triterpenes, phenols, and tannins using methods suggested by Roopashree et al. (2008) [36]. The acid butanol test was used to determine whether the tannins were of the condensed type [37].

### 2.3. Bacterial Strains

Three biomonitor strains were used for anti-AHL activity:

- *Chromobacterium violaceum* ATCC 12472, wild type strain that produces a purple pigment in response to QS (C6 AHL) molecules that it produces;
- *Chromobacterium violaceum* ATCC 31532, nonpigmenting strain that overproduces C6-AHL, the autoinducer;
- *Chromobacterium violaceum* 026, a mini Tn5 mutant of* Chromobacterium violaceum *31532, which is unable to produce the autoinducer C6-AHL but responds to an exogenous supply of C6-AHL by producing the purple pigment [38].

All three strains were maintained on Luria agar.

Biomonitor strain* Staphylococcus aureus agr*P3::*blaZ* RN6390 pRN8826 that contains* agr*P3-*blaZ* fusion plasmid and produces *β*-lactamase spontaneously due to* agr* expression was used for detection of anti-QS activity that targeted AIP mediated QS. This strain was maintained on Luria agar with 10 *μ*g/mL chloramphenicol.

### 2.4. Determination of Minimum Inhibitory Concentration (MIC) of Extracts against Biomonitor Strains

MICs of the extracts were determined essentially to decide subinhibitory concentrations (SICs) that were used for quantitative anti-QS activity in Gram positive and Gram negative biosensor strains. MIC was determined using the microdilution method given by Valgas et al. (2007) [39]. 8 mg/mL of methanolic extract in 7.8% DMSO of each was diluted twofold in Luria broth to a total volume of 100 *μ*L in 96-well microtitre plates. Wells were inoculated with 5 *μ*L of a bacterial suspension (10^8^ CFU/mL) of the* C. violaceum* 12472/*S. aureus agr*P3::*blaZ* RN6390. All experiments were performed in triplicate and plates were incubated at 37°C for 24 h. After incubation 50 *μ*L of 0.2 mg/mL alcoholic solution of INT (2-(4-iodophenyl)-3-(4-nitrophenyl)-5-phenyl-2H-tetrazolium) (Hi media) was added and plates were incubated at 37°C for 30 min. Reduction of INT to its formazan product was a clear indication of growth/no growth. Change in color to pink was noted as growth. Concentrations lower than the cidal concentration were then used as SICs.

### 2.5. Anti-QS Activity of Plant Extracts Using Gram Negative Biomonitor Strain

#### 2.5.1. Quantitative Studies

For quantitative studies of the anti-QS activity, a modified method of Blosser and Gray (2000) [40] was used. This assay estimated the pigment produced by* C. violaceum* ATCC 12472. Concentrations of the extracts that were selected were below the MIC and equaled 0.5MIC (*N*/2), 0.25MIC (*N*/4), 0.125MIC (*N*/8), 0.0625MIC (*N*/16), and 0.03125MIC (*N*/32) of each extract and were prepared in Luria broth. To 1 mL of each SIC 50 *μ*L of overnight culture (10^7^ cfu/mL) of* C. violaceum* ATCC 12472 was added and tubes were incubated for 24 hours at room temperature. Medium without the extract served as a positive control, medium with 7.8% DMSO served as vehicle control, and medium with subinhibitory concentrations of extract but no culture served as blanks. After incubation, amount of growth in each tube was accessed by measuring absorbance at 655 nm. The pigment produced was extracted by adding equal quantity of acetone to the harvested biomass [41] and absorbance was measured at 567 nm on Jasco V363 spectrophotometer against each blank. Percentage of pigment formed was calculated with respect to the vehicle control. All assays were performed in triplicate and average percent pigmentation values were reported with SD that was calculated using the Microsoft EXCEL software. Minimum concentration in mg/mL of plant extract, at which 50% reduction in pigment formation was observed, was designated as the quorum sensing inhibitory concentration (MQSIC) [31].

#### 2.5.2. Determination of* luxR*/*luxI* Effect

To determine whether inhibition of QS was due to the effect on* N*-acyl homoserine lactone response (*luxR* effect) or due to its effect on synthesis of AHL (*luxI* effect), method of Mihalik et al. (2008) [42] was used. Discs of Whatman filter paper number 1 with 75 *μ*L of methanolic plant extracts were placed on Luria agar plates.* C. violaceum* strains, CV026 and CV31532, were streaked in circles 8–10 mm apart such that in one plate strain CV026 formed the inner circle and strain CV31532 the outer circle, while in the other plate it was vice versa. On incubation at RT for 24 hours pigmentation in the CV026 strain was used to determine the* luxR*/*luxI* effect. A lowered signal in the form of pigmentation of CV026 when it forms the outer circle suggested that LuxI was the target while a lowered signal in the form of pigmentation of CV026 when it forms the inner circle suggested that LuxR was the target.

### 2.6. Anti-QS Activity of Plant Extracts Using Gram Positive Biosensor Strain

Quantitative measurement of* agr* activity was done using the* S. aureus agr*P3::*blaZ* reporter assay, originally described by Ji et al. (1995) [43]. This assay measured RNA III production in the form of *β*-lactamase activity using the chromogenic cephalosporin, nitrocefin, as a substrate.* S. aureus agr*P3-*blaZ* strain was grown in CYGP broth overnight at 37°C. The overnight broth culture was further diluted 1/100 with fresh medium and incubated at 37°C to attain logarithmic phase (*A*_600_, 0.4). In a microtitre plate, 5 *μ*L of tannin-rich plant extract prepared in CYGP broth was added to 45 *μ*L of log phase culture such that it reached a subinhibitory concentration equivalent to 0.5MIC (*N*/2), 0.25MIC (*N*/4), 0.125MIC (*N*/8), 0.0625MIC (*N*/16), and 0.03125MIC (*N*/32). In the positive control well 5 *μ*L of supernatant of the overnight broth culture was added as a source of AIP to 45 *μ*L of the log phase culture, whereas 5 *μ*L of 7.8% DMSO was added to 45 *μ*L of the log phase culture as vehicle control. The plate was incubated at 37°C on Selec microplate shaker for 55 minutes. After incubation 50 *μ*L of CYGP broth containing 5 mM sodium azide was added, followed by 50 *μ*L of 132 *μ*g/mL nitrocefin (Toku-E) prepared in 0.1 M sodium phosphate buffer, pH 5.8. *β*-lactamase activity was measured in Biorad imark microplate reader using kinetic mode at *ε*490–*ε*650 nm. One unit of *β*-lactamase activity was defined as an increase in 0.001 OD units per min. All assays were performed in triplicate and average percent enzyme activity was calculated with respect to the vehicle control. % enzyme activity values were reported with SD that was calculated using the Microsoft EXCEL software. Minimum concentration of extract in mg/mL that showed 50% inhibition in activity was determined and designated as the minimum quorum sensing inhibitory concentration (MQSIC) [32].

## 3. Results and Discussion

### 3.1. Phytochemical Screening

All the methanolic extracts when screened for their phytochemical constituents showed strong reactions with FeCl_3_. The protein precipitation test too was strongly positive for all extracts. This confirmed that all the shortlisted plants were rich in tannins. Tannins are primarily of two types: condensed and hydrolysable. Qualitative analysis for condensed tannins showed that the extracts of* Acacia arabica*,* Terminalia arjuna*,* Casuarina equisetifolia*,* Thespesia populnea*,* Mangifera indica* (seed kernel), and* Embelia ribes* were rich in condensed tannins. In contrast, extracts of* Phyllanthus emblica*,* Terminalia bellirica*,* Terminalia chebula*,* Punica granatum* and* Mangifera indica* (flower), and* Syzygium cumini* were rich in hydrolysable tannins as they showed high tannin content but gave a negative acid butanol test [44–49].

### 3.2. Anti-QS Activity of Plant Extracts Using Gram Negative Biomonitor Strain (Anti-AHL Activity)

#### 3.2.1. Quantitative Studies

**Figure 1:**
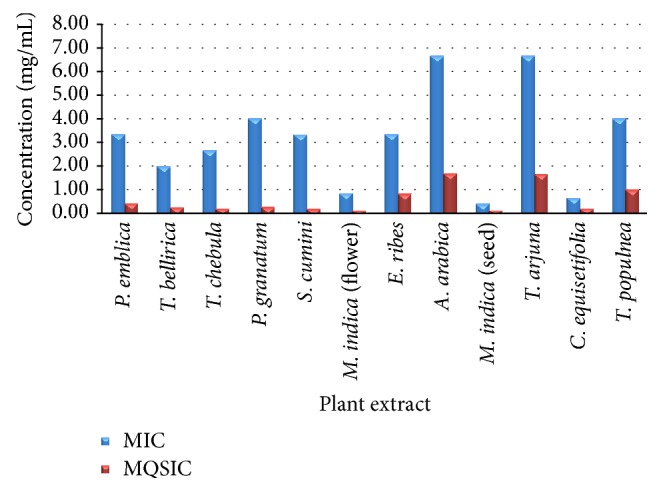
Comparison of MIC values with MQSIC values for anti-quorum sensing activity of plant extracts, detected using biosensor strain* C. violaceum* 12472.

Amount of pigment produced by* C. violaceum* 12472 on exposure to subinhibitory concentrations of extracts showed an increase with decreasing concentration of extract. All the twelve extracts showed anti-QS activity at subinhibitory concentrations (Table 1). All the extracts showed not less than 65% growth in* N*/2 concentration, not less than 82% in* N*/4 concentration, and between 85 and 114% thereafter with respect to the vehicle control, indicating that the SICs used did not significantly affect cell density. A comparison of MQSIC with the MIC of the extracts (Figure 1), therefore, clearly depicted the range of concentration where the extracts had a specific and optimal QS inhibition activity with zero or minimal effect on growth of* C. violaceum*. Extracts of* P. granatum*,* S. cumini*, and* T. chebula* were most significant as there was a marked difference (1/16) in their MIC and MQSIC. In contrast, extracts of* E. ribes*,* A. arabica* and* M. indica* (seed),* T. arjuna*,* C. equisetifolia*, and* T. populnea* had a very low range of concentration at which they could prevent QS, as the difference between their MIC and MQSIC was only 4-fold, while extracts of* P. emblica*,* T. bellirica*, and* M. indica* showed* N*/8 as their MQSIC value.

#### 3.2.2. *luxI*/*luxR* Effect

**Figure 2:**
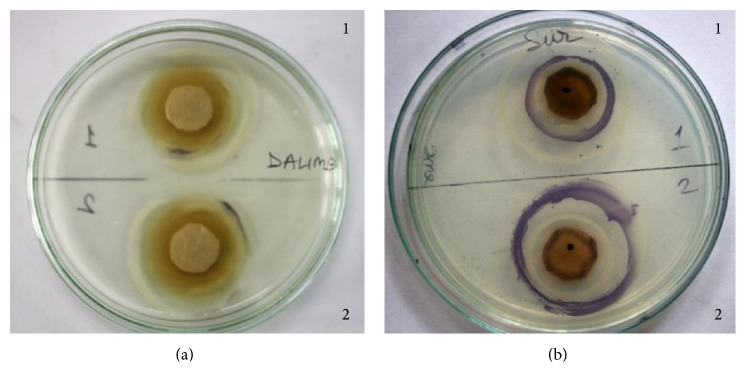
*luxI* and* luxR* effect: extract prepared from* P. granatum* affects production as well as activity of the autoinducer (a). Extract prepared from* C. equisetifolia* is unable to affect either production or binding of the autoinducer (b) (1:* luxR* effect, 2:* luxI* effect).

To investigate whether QS inhibition might be due to interference with acyl homoserine lactone production (*luxI* effect) or a transcription response (*luxR effect*) the* luxI/luxR* assay was done. Amongst the hydrolysable tannin-rich extracts studied* T. bellirica*,* T. chebula*,* S. cumini*, and* M. indica* (seed) showed a putative* luxI* effect, indicating that they would affect production of the C6 HSL by CV31532, whereas* P. emblica* showed a putative* luxR* effect indicating that it would interfere with the binding of the autoinducer to receptors on CV026. It is interesting to note that* P. granatum* and* M. indica* (flower) showed both* luxI* and* luxR* effect (Table 2 and Figure 2).

### 3.3. Anti-QS Activity of Plant Extracts Using Gram Positive Biosensor Strain (Anti-*agr* Activity)

**Figure 3:**
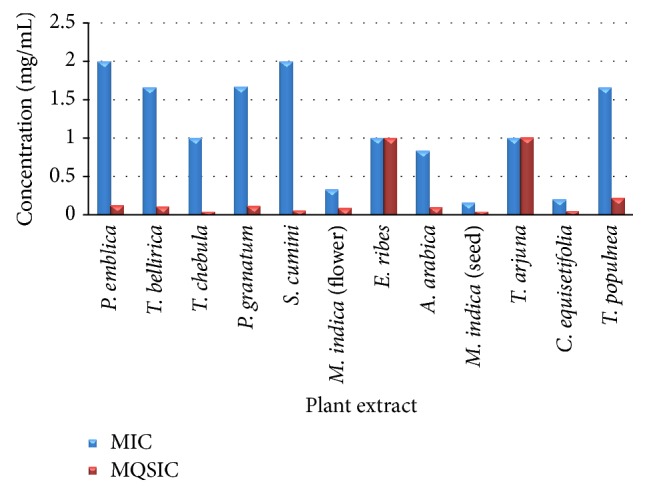
Comparison of MIC values with MQSIC values for anti-quorum sensing activity of plant extracts, detected using biosensor strain* S. aureus agr*P3::*blaZ*.

*β*-lactamase produced as a result of QS in biomonitor strain* S. aureus agr*P3::*blaZ* RN6390 pRN8826 was estimated using chromogenic cephalosporin that on breakdown by *β*-lactamase gave a red colored product. Estimation of the enzyme units of *β* lactamase was used as a measure of* agr* activity and, hence, QS in the* S. aureus agr*P3::*blaZ* strain (Table 3). The most significant extract amongst those tested was* T. chebula* as it showed only about 30% activity at the lowest subinhibitory concentration tested that is 0.03125 of its MIC.* S. cumini*,* P. granatum*, and* M. indica* (seed) too showed low activity at this concentration. A comparison of the MQSIC with the MIC of the extracts (Figure 3) was used as a representation of the range of concentration, where the extracts had a specific and optimal QS inhibition activity with zero or minimal effect on growth of* S. aureus agr*P3::*blaZ* RN6390. The MQSIC in* P. emblica*,* T. bellirica*,* P. granatum*, and* M. indica* (seed) was 16 times lower than its MIC and in* T. populnea* and* A. arabica* it was found to be 8 times lesser, while in* C. equisetifolia* it was 4 times lesser than its MIC.

In a qualitative study, Shukla and Bhathena (2014) [50] have reported that several tannin-rich crude extracts show a broad spectrum of anti-QS activity. Another qualitative study on anti-AHL activity of pericarp of* Punica granatum* and an ethyl acetate fraction of* S. cumini* leaves has also been reported [22, 51]. Hasan et al. (2012) [23] have reported that* P. emblica* fruit extract can suppress quorum sensing genes in* S. mutans*. These reports draw attention to the fact that tannin-rich extracts of medicinal plants influence quorum sensing mechanisms.

The current study is a quantitative evaluation of anti-AHL and anti-*agr* activity of tannin-rich plants. One of the striking inferences of this study is that extracts rich in condensed tannins are unable to prevent AHL as well as* agr* mediated bacterial communication over a wide range of subinhibitory concentrations. On the other hand, extracts rich in hydrolysable tannins, namely,* P. emblica*,* T. bellirica*,* T. chebula*,* P. granatum*,* S. cumini*, and* M. indica* (flower), exhibit a broad spectrum anti-QS activity, that is, affecting activity of acyl homoserine lactones as well as autoinducers over a wide range of subinhibitory concentrations. Okuda and Ito (2011) [52] have attributed most of the biological and pharmacological activities of tannins determined so far to the simpler tannins like hydrolysable tannins. Among the extracts rich in hydrolysable tannins,* T. chebula*,* P. granatum*, and* S. cumini* can be categorized as potent quorum sensing inhibitors as they demonstrate this activity at concentrations that are several times lower than their respective MIC values. Anti-AHL activity of pericarp of* Punica granatum* using a qualitative method was reported by Zahin et al. (2010) [22]. The current study goes one step further to show that* P. granatum* is effective over a wide range of concentrations much lesser than its MIC and also that it affects AHL activity by influencing both production and its binding. The anti-QS property of* P. granatum* gets augmented, as it also shows significant anti-*agr* activity. Since all the extracts show distinct protein binding ability, these extracts may be disrupting QS either by inactivating enzymes responsible for the synthesis of the autoinducers or by binding to protein receptors of QS signals as demonstrated in the* luxI/luxR* assay.

## 4. Conclusion

Although the discovery of antibiotics has been a boon to human civilization, the current traditional antimicrobials need to be reviewed for their efficacy because of the increasing occurrence of multidrug resistant strains. One novel therapeutic approach to overcome the problem of resistance is the use of antipathogenic drugs that target key regulatory bacterial systems responsible for the expression of virulence factors. Since quorum sensing leads to expression of virulence genes in several known pathogens, agents that can interrupt bacterial communication can be used as antipathogenic drugs. The study on tannin-rich crude extracts from Indian medicinal plants used in Ayurveda showed that extracts of hydrolysable tannin-rich plants,* Phyllanthus emblica*,* Terminalia bellirica*,* Terminalia chebula*,* Punica granatum*,* Syzygium cumini*, and* Mangifera indica* (flower), are effective QSIs that can interrupt QS in both Gram positive and Gram negative bacteria over a wide range of subinhibitory concentrations, suggesting the presence of a high concentration of anti-QS molecules. Thus plant extracts rich in hydrolysable tannins can be used as broad spectrum antipathogenic drugs at concentrations that will not impose selection pressure. As this study has been done on crude extracts, purification and identification of the active principle along with verification of its anti-QS activity using* in vivo* studies are essential. However, such a pharmacognosy approach to the so-called alternative systems of medicine can be of immense help in the search for novel phytocompounds with medical applications.

## Figures and Tables

**Table 1:** Quantitative studies of anti-quorum sensing activity detected as pigmentation in *C. violaceum *ATCC 12472 in the presence of subinhibitory concentrations of plant extracts.

Plant	% pigmentation with respect to untreated control (*N* = MIC in mg/mL)				
	*N*/2	*N*/4	*N*/8	*N*/16	*N*/32
*P. emblica*	7.29 ± 12.36	29.58 ± 8.66	41.70 ± 6.19	66.50 ± 2.82	102.84 ± 8.94
*T. bellirica*	0.57 ± 1.00	19.66 ± 8.79	28.54 ± 8.9	56.63 ± 6.01	68.39 ± 12.13
*T. chebula*	0.00	2.02 ± 2.93	21.22 ± 4.61	47.85 ± 5.40	72.18 ± 9.45
*P. granatum*	20.08 ± 6.78	23.21 ± 7.86	25.75 ± 8.54	32.17 ± 3.49	72.05 ± 11.26
*S. cumini*	0.72 ± 0.66	5.64 ± 5.32	12.88 ± 7.72	28.70 ± 6.86	78.53 ± 19.27
*M. indica *(flower)	6.48 ± 0.42	28.59 ± 12.61	34.19 ± 12.08	72.33 ± 6.49	87.05 ± 8.43
*E. ribes*	13.34 ± 6.21	30.60 ± 6.02	51.19 ± 3.83	89.49 ± 14.54	90.18 ± 4.00
*A. arabica*	5.56 ± 3.52	42.50 ± 7.11	56.68 ± 2.25	109.21 ± 31.91	119.58 ± 13.93
*M. indica *(seed)	4.83 ± 1.76	47.05 ± 3.20	61.47 ± 8.58	76.55 ± 17.50	87.87 ± 4.97
*T. arjuna*	3.20 ± 2.86	46.26 ± 4.52	71.40 ± 15.54	88.89 ± 15.45	96.54 ± 3.83
*C. equisetifolia*	6.93 ± 2.54	16.85 ± 3.72	59.94 ± 10.98	89.43 ± 11.55	86.55 ± 13.10
*T. populnea*	0.01 ± 0.01	10.14 ± 9.74	56.16 ± 9.55	68.72 ± 11.66	80.09 ± 8.92

**Table 2:** Demonstration of *luxI*/*luxR* effect by plant extracts.

Extract	*luxI* effect	*luxR* effect
*P. emblica*	−	+
*T. bellirica*	+	−
*T. chebula*	+	−
*P. granatum*	+	+
*S. cumini*	+	−
*M. indica *(flower)	+	+
*E. ribes*	−	−
*A. arabica*	−	−
*M. indica *(seed)	+	−
*T. arjuna*	−	−
*C. equisetifolia*	−	−
*T. populnea*	−	−

**Table 3:** Quantitative studies of anti-quorum sensing activity detected as *β*-lactamase activity in biosensor strain *S. aureus agr*P3::*blaZ* in the presence of subinhibitory concentrations of plant extracts.

Plant	% *β*-lactamase activity with respect to untreated control (*N* = MIC in mg/mL)				
	*N*/2	*N*/4	*N*/8	*N*/16	*N*/32
*P. emblica*	20.9 ± 7.87	37.9 ± 2.43	34.5 ± 3.25	39.1 ± 2.47	63.6 ± 13.08
*T. bellirica*	16.3 ± 2.45	5.6 ± 2.11	36.9 ± 3.55	43.5 ± 4.33	80.2 ± 6.32
*T. chebula*	7.5 ± 5.85	18.9 ± 2.69	24.7 ± 5.70	28.4 ± 9.48	30.2 ± 4.18
*P. granatum*	14.6 ± 5.13	21.3 ± 2.54	20.8 ± 6.26	33.2 ± 7.79	51.1 ± 11.24
*S. cumini*	6.5 ± 2.12	22.4 ± 0.77	28.1 ± 5.06	39.6 ± 5.25	46.0 ± 4.07
*M. indica *(flower)	0.0	0.0	80.3 ± 9.71	82.7 ± 10.71	82.3 ± 14.53
*E. ribes*	57.4 ± 16.45	65.8 ± 5.28	65.0 ± 7.00	70.3 ± 9.27	97.7 ± 13.19
*A. arabica*	33.0 ± 1.97	32.5 ± 6.34	34.5 ± 4.61	55.2 ± 3.72	61.8 ± 2.65
*M. indica *(seed)	0.0	28.4 ± 10.25	53.8 ± 2.27	52.4 ± 4.77	54.6 ± 1.87
*T. arjuna*	52.6 ± 9.95	53.7 ± 15.32	50.2 ± 1.64	52.3 ± 3.46	60.6 ± 6.83
*C. equisetifolia*	37.0 ± 8.53	38.3 ± 7.22	59.7 ± 2.46	64.8 ± 9.49	64.4 ± 6.56
*T. populnea*	13.0 ± 8.80	22.7 ± 2.84	38.5 ± 3.59	55.5 ± 1.22	57.8 ± 6.28
